# Using personalized medicine in gliomas: a genomic approach to diagnosis and overcoming treatment resistance in a case with pleomorphic xanthoastrocytoma

**DOI:** 10.1007/s00415-019-09575-8

**Published:** 2019-11-21

**Authors:** Yolanda Piña, Michael J. Fusco, Robert J. Macaulay, Christine M. Walko, Edwin Peguero, Brittany R. Evernden, Keiran S. Smalley, Peter Forsyth

**Affiliations:** grid.468198.a 0000 0000 9891 5233 H. Lee Moffitt Cancer Center & Research Institute, 12902 USF Magnolia Dr., Tampa, FL 33617 USA

**Keywords:** Pleomorphic xanthoastrocytoma, *BRAF* inhibition, *MEK* inhibition, V600E mutation, Autophagy inhibition, Chloroquine

## Abstract

**Introduction:**

A patient who was initially considered to have a glioblastoma (GBM) had molecular analysis, showing that it was a pleomorphic xanthoastrocytoma (PXA). Up to 78% of PXA tumors have *BRAF* V600E mutations. Primary brain tumors with *BRAF* mutations can have a good response to *BRAF* MEK inhibitors (BRAF MEKi), and there may be a synergistic response when combined with autophagy inhibitors.

**Presentation of the case:**

A 20-year-old man found to have a large brain mass with midline shift underwent resection. He was diagnosed with “GBM” and treated with radiation and temozolomide with subsequent disease recurrence. Review of histology showed malignant PXA with *BRAF* V600E mutation. Treatment with Dabrafenib and Trametinib was started, and tumor size increased in size after 14 months of treatment. Given studies showing that resistance to *BRAF* inhibition can be overcome by autophagy inhibition, chloroquine was added. Patient has been on “triple” therapy for 15 months and has radiographically Stable Disease. At MCC, 3% of patients with gliomas have *BRAF* mutations who could potentially benefit from this combination therapy.

**Conclusion:**

This is the first report of a PXA patient receiving therapy with BRAF MEKi and an autophagy inhibitor with prolonged stable disease. This patient highlights the importance of a molecular interrogation in gliomas to provide an integrated diagnosis and effective treatment. This may be useful in up to 3% of glioma patients with *BRAF* mutations. Molecular testing in neuro-oncology is providing new avenues of diagnosis and treatment, and detailed molecular interrogation should be considered routine.

## Introduction

Pleomorphic xanthoastrocytoma (PXA) is a rare low-grade astrocytoma, which accounts for less than 1% of all central nervous system (CNS) neoplasms. It is most commonly found in children and young adults. It is characterized by spindle-shaped or pleomorphic astrocytes with frequent intracytoplasmic lipid vacuoles, moderate-to-marked nuclear atypia, eosinophilic granular bodies, frequent desmoplasia, and patchy chronic inflammation. Mitotic activity is usually sparse. PXA is usually low grade, but may be anaplastic as in the current case report. Recently, a growing body of evidence has shifted the classification of gliomas based on histological and molecular findings, with PXA and anaplastic PXA perceived as separate entities, and classified by the World Health Organization (WHO) as grade II and III, respectively. This is mainly based on the mitotic index (MI), with WHO grade III based on MI equal to or greater than 5 mitotic cells per every 10 high power field (HPF), with or without accompanying necrosis [1, 2]. Magnetic Resonance Imaging (MRI) of the brain demonstrates either a solid mass or a solid-cystic pattern with the cystic component hypointense on T1-weighted images and hyperintense on T2, and the solid component showing contrast enhancement that is hypo- or isointense on T1-weighted images and iso- or slightly hyperintense on T2 [3, 4].

Sixty to seventy-eight percent of PXA tumors have a *BRAF* V600E mutation. This mutation is frequently found in PXA and has allowed targeted molecular therapy in many other different tumor types [5–10]. There are few clinical trials in *BRAF*-mutated gliomas. The VE-BASKET study, which treated a wide range of glioma patients with *BRAF* V600 mutation with *BRAF* inhibition, showed a PXA case with a complete response (14% of PXA treated, *n* = 7, and 4% of all gliomas, *n* = 24), two cases with partial responses (29% of PXA, and 8% of all gliomas), and three cases with stable disease (43% of PXA, and 12.5% of all gliomas). The median progression-free survival was 5.5 months in all the gliomas treated, and more than 39.1 months in a PXA case [11]. There are several case reports of combined BRAF MEKi in PXA patients [12–14]. As well as an enhanced response to *BRAF* inhibition when combined with autophagy inhibition in glioma cell lines [15]. However, experience with BRAF MEKi with the addition of chloroquine has not been published in PXAs. Here, we present a patient with a malignant PXA with a *BRAF* V600E mutation, who had a prolonged response to BRAF MEKi and benefited by the addition of chloroquine with an ongoing prolonged disease control.

## Case presentation

**Fig. 1:**
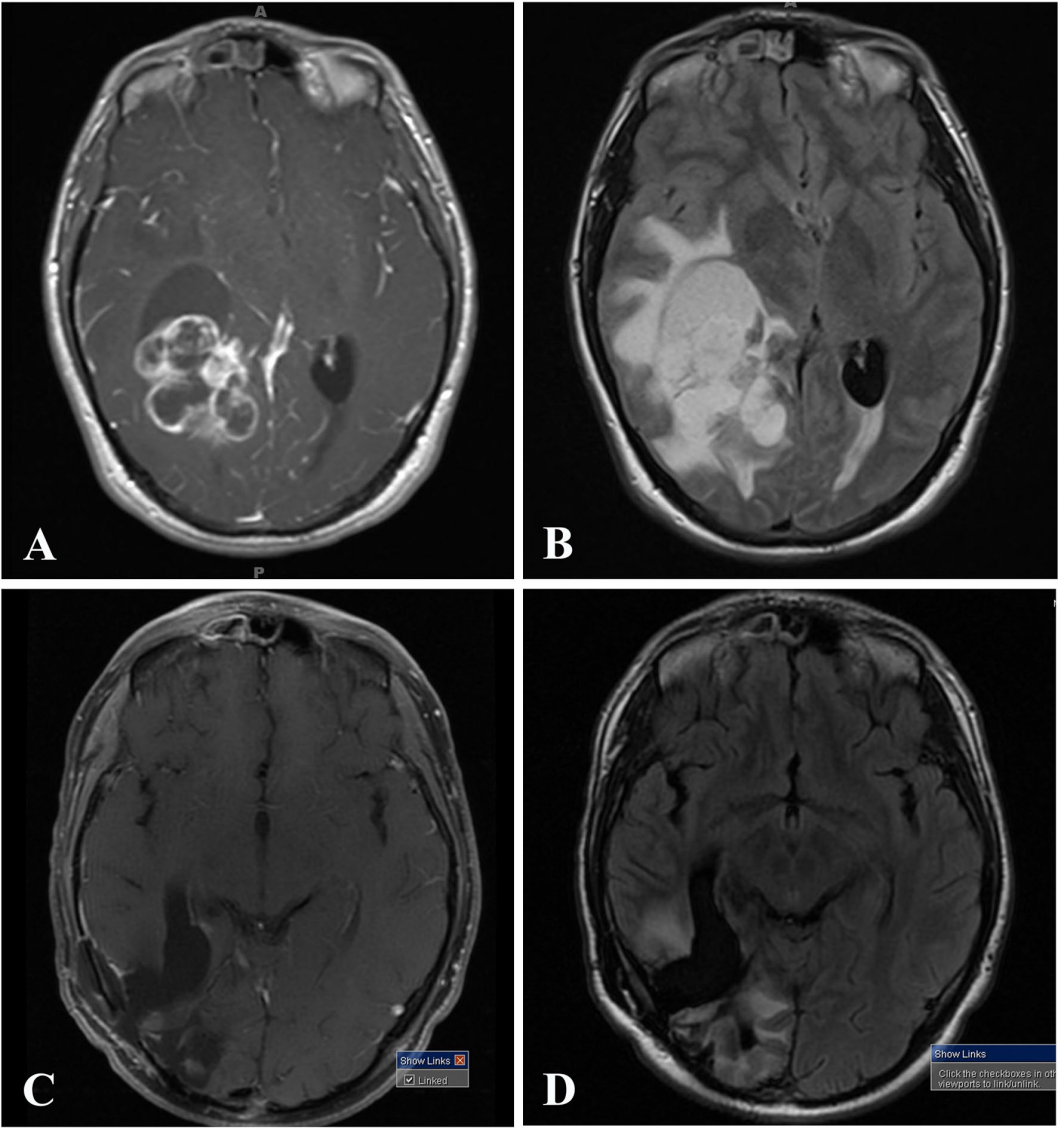
MRI of the brain demonstrating a right-sided, large parieto-temporo-occipital mass, which appeared unusual for a classical GBM, with surrounding vasogenic edema and a right-to-left midline shift. **a**, **b** Initial MRI of the brain prior to surgery for tumor resection in January 2015. **a** T1-weighted post-contrast demonstrating heterogeneous enhancement. **b** T2-weighted fluid-attenuated inversion recovery (FLAIR) shows significant surrounding vasogenic edema. **c**, **d** Status-post resection and two cycles of maintenance TMZ in September 2015. **c** T1-weighted post-contrast. **d** Status-post resection, T2-weighted FLAIR sequence

A 19-year-old man developed blurry vision with new headaches in November 2014. He had bilateral papilledema. A MRI brain showed a large right-sided lesion involving the parieto-temporal lobes, hyperintense on T1 and T2-weighted sequences, with significant surrounding vasogenic edema on T2-weighted fluid-attenuated inversion recovery (FLAIR), contrast enhancement post-gadolinium, and a right-to-left midline shift (Fig. 1a, b). The overall appearance of this lesion looked a bit unusual for a classical GBM. He had a subtotal resection on January 30th, 2015, and was diagnosed by a local pathologist with a “GBM”. He completed 6 weeks of radiation therapy (RT) and temozolomide (TMZ). Four months later, a follow-up MRI showed an increase in the size of the enhancing tumor and, despite the possibility of pseudoprogression, a second surgical resection was performed on June 2nd, 2015 and showed “GBM”. Maintenance TMZ was started and follow-up imaging showed stable disease (Fig. 1).

**Fig. 2:**
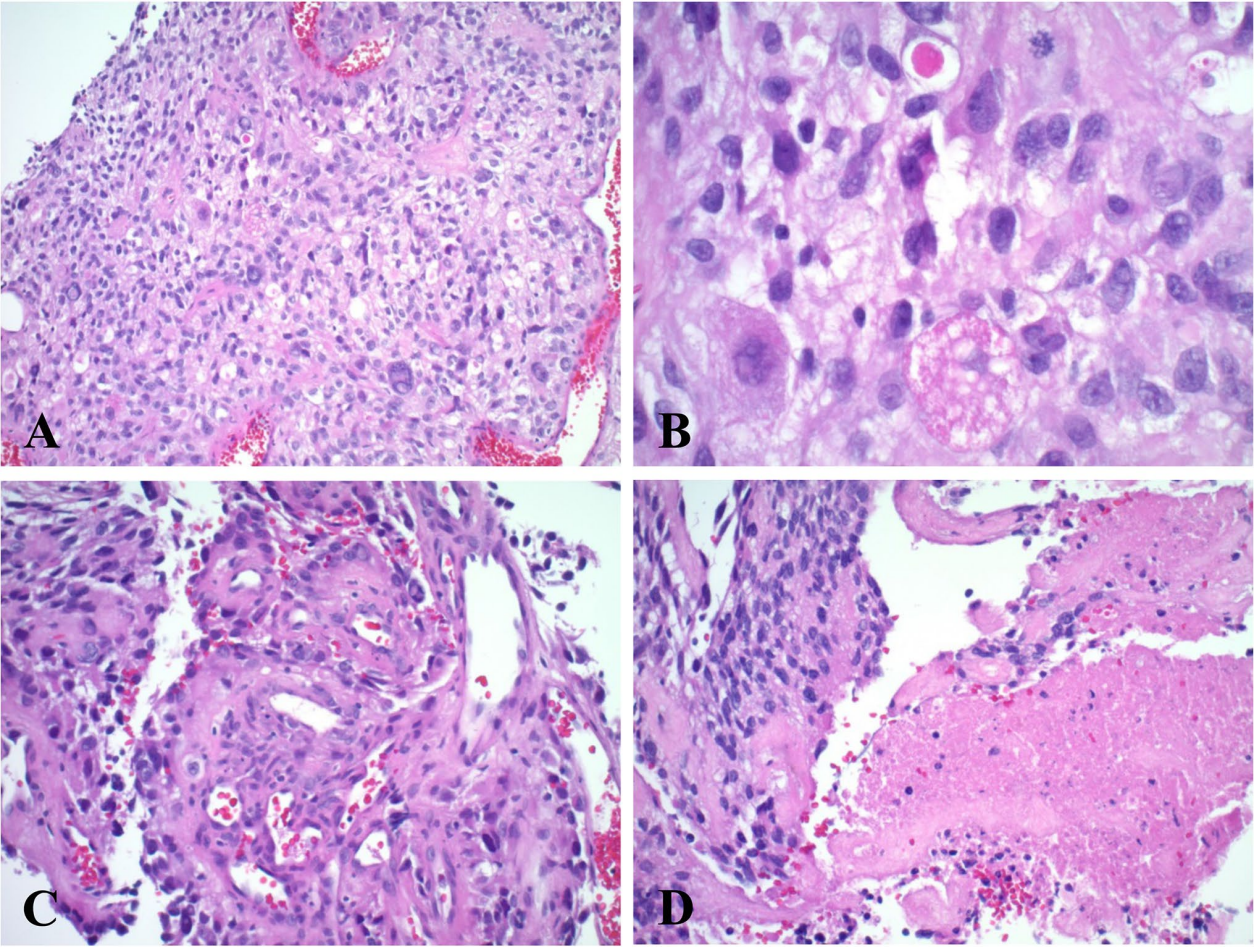
Histology of tumor sample demonstrating an atypical malignant glioma that resembled a PXA rather than a GBM. **a** Non-infiltrating tumor, WHO grade III–IV, H&E, × 20 HPF. **b** Neoplastic astrocytes and binucleated giant cells, with prominent nucleoli, nuclear vacuolation, and eosinophilic granular bodies, H&E, × 600 HPF. **c** Vascular proliferation, × 200 HPF. **d** Necrosis, × 200 HPF

**Fig. 3:**
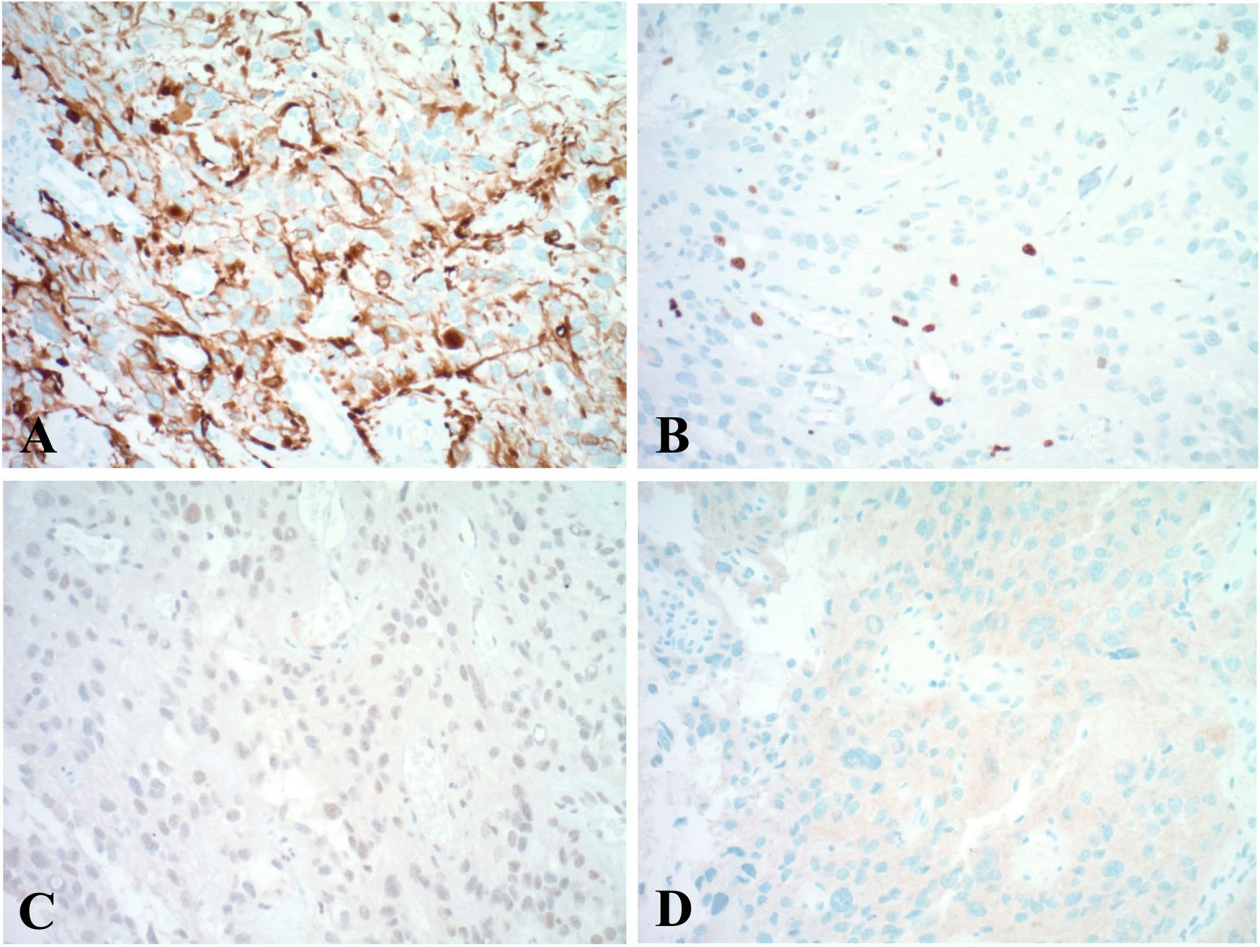
Immunohistochemistry (original magnification × 200 HPF). Tumor cells: **a** were GFAP-positive; **b** had a Ki-67 proliferation index of 2%; **c** retained ATRX; and **d** were diffusely positive for *BRAF* V600E

The patient was referred to the Neuro-Oncology clinic at MCC in June 2015. Histology review showed that he had a malignant PXA grade III–IV, rather than a GBM. It had multinucleated giant cells, prominent nucleoli, and eosinophilic granular bodies on 600 × HPF, and a high mitotic index with dysplastic neurons on 200 × HPF (Fig. 2). Histological samples were GFAP positive, with necrosis, ATRX retained, had a proliferation rate of 2% by Ki-67, and was positive for *BRAF* V600E on IHC (Fig. 3). Foundation one testing confirmed the *BRAF* V600E mutation, IDH1 wild-type, and no EGFRviii. Other testing showed that the tumor was negative for 1p/19q co-deletion and was O6-methylguanine-DNA methyltransferase (MGMT) promoter unmethylated.

**Fig. 4:**
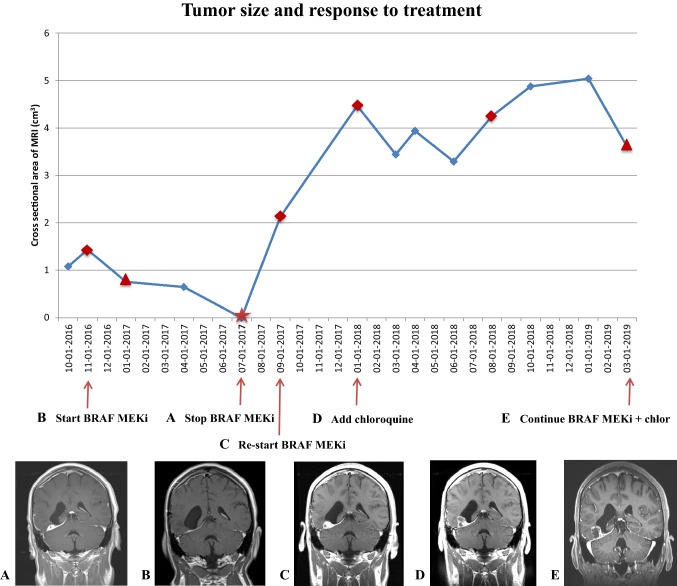
Lesion size changes based on RANO criteria and response to treatment. **a** Dabrafenib and trametinib combination therapy was initiated. **b** Treatment was discontinued for a holiday and re-started after disease progression (**c**). **d** Autophagy inhibitor was added to dabrafenib and trametinib. **e** Last MRI brain showed slight decrease in the size of the mass and the central cystic component measuring 1.9 × 1.6 cm, compared to prior measuring 2.1 × 1.5 cm 2 months earlier; there were no changes in the peripheral enhancing aspect of the lesion. Target lesion response measured by RANO criteria. Star = CR (all target lesions disappeared). Triangle = SD (SPD < 50% decrease to < 25% increase). Rhomboid = PD (SPD increased by ≥ 25% from nadir value)

After an initial 17 months of stable disease, on his MRI, there was a small increase in the size of his tumor (Fig. 4). Accordingly, combination therapy with *BRAF* kinase and MEK inhibitors, Dabrafenib 150 mg PO BID and Trametinib 2 mg PO OD, was started on November 2016. As soon as 2 months after starting treatment, there was radiographic evidence of disease regression, though it did not meet the criteria for a Partial Response because of its small size. The patient was continued on this treatment regimen for 10 months and further serial imaging showed stable disease.

After 8 months of treatment, in July 2017, treatment was held to give the patient a “drug holiday,” but, 2 months later, his MRI showed disease progression. Dabrafenib and Trametinib were re-started, and he remained stable until January 2018 when he had disease progression with BRAF MEKi. Since resistance to *BRAF* inhibition can be overcome by autophagy inhibition [15–17], we added the autophagy inhibitor chloroquine (500 mg PO daily) to his BRAF MEKi therapy. Each tablet of chloroquine contains 500 mg of chloroquine phosphate USP and the equivalent to 300 mg chloroquine base, which is the standard, maximal safe dose that is FDA-approved for adults [18].

Based on the Response Assessment in Neuro-Oncology (RANO) criteria, the lesion size was measured, the sum of the perpendicular diameters (SPD) calculated and plotted (Fig. 4). The tumor decreased by more than 25% after BRAF MEKi was started (Fig. 4a) but unfortunately increased after a drug holiday (Fig. 4c), and continued to grow despite re-starting therapy with BRAF MEKi (Fig. 4d), at which point the autophagy inhibitor chloroquine was added halting the rate of tumor progression and even causing a slight decrease in the lesion size (Fig. 4e).

There are no reported potential interactions between chloroquine and Dabrafenib and/or Trametinib. Chloroquine’s adverse effects can be multisystemic affecting the eyes (e.g., retinopathy, visual disturbances), hearing, liver, gastrointestinal system (e.g., nausea, vomiting, diarrhea, abdominal cramps), muscles (e.g., myopathy), skin (e.g., erythema multiforme, Stevens–Johnson syndrome), cardiac (e.g., prolonged QT interval), hematologic system (e.g., pancytopenia), and nervous system (e.g., seizures, extrapyramidal signs) [18]. Given these side effects, we had taken precautionary measures with close monitoring every 1–2 months since started triple therapy, checking complete blood cells counts, complete metabolic panels, electrocardiogram, and echocardiograms. Overall, our patient tolerated the triple therapy well for 17 months until recently, when he complained of mild nausea, diarrhea, and a skin rash. The decision was made to hold chloroquine, while continuing Dabrafenib and Trametinib, with plans to re-assess him in 2 months.

In summary, radiographically, he has had Stable Disease with BRAF MEKi for 14 months, and later with the addition of chloroquine for a total of > 2.5 years of treatment (triple therapy for 17 months), without major side effects from the treatment, until recently for which he is receiving a drug holiday from chloroquine.

## Discussion

PXA is a rare low-grade astrocytoma, which may be anaplastic, as in the case herein presented. An MRI can show either a solid mass or a solid-cystic lesion, with the cystic component being hypointense on T1 and hyperintense on T2, and the solid component having contrast enhancement that is hypo- or isointense on T1 and iso- or slightly hyperintense on T2 [3, 4]. These radiographic findings make it possible to misdiagnose this as a malignant glioma or a GBM. Histologically, PXA is composed of neoplastic astrocytes and multinucleated giant cells with prominent nucleoli and/or nuclear vacuolation, with immunoreactivity to S100 protein and GFAP [19]. Sixty to seventy-eight percent of PXA tumors have been found to carry *BRAF* V600E mutation, which was more frequently found in PXA tumors than in any other neuroepithelial neoplasm of the CNS [5–10]; it can be detected via immunohistochemistry [20] or by molecular techniques. The relationship of anaplastic PXA to epithelioid glioblastomas, which also carry the *BRAF* V600E alteration, remains unsettled.

*BRAF* V600E mutations result in the constitutive activation of the *BRAF* pathway, which includes mitogen-activated extracellular signal kinase (MEK) 1 and 2 activation. This mutation is found in a number of primary brain gliomas, including PXAs [7, 9, 21, 22], gangliogliomas, and papillary craniopharyngiomas [23]. Dabrafenib (Tanfinlar^®^) is a *BRAF* kinase inhibitor approved by the U.S. FDA for *BRAF* V600E melanomas [24]. Metastatic melanoma tumors with *BRAF* V600E mutations have a complete (6%) or partial tumor regression (62.5%) in most patients treated with the *BRAF* inhibitor [25]. Combination therapy with Dabrafenib and Trametinib (Mekinist^®^), an MEK 1 and 2 inhibitor, produces superior response rate to BRAF inhibition alone and has been approved for metastatic melanoma with either *BRAF* V600E or V600K mutations [26].

Several Clinical Trials have shown that *BRAF* inhibition monotherapy (e.g., vemurafenib) is effective in melanoma brain metastases [27–30] and small case series have shown that several primary *BRAF* mutant brain tumors (i.e., primary neuroepithelial brain tumors, malignant astrocytomas, papillary craniopharyngiomas, and other nonmelanoma cancers) also respond to *BRAF* inhibition [21, 23, 31, 32]*.* Surprisingly, papillary craniopharyngiomas have *BRAF* mutations and patients may respond dramatically [23]. Others have reported *BRAF* mutant anaplastic PXAs having partial responses to *BRAF* inhibitor monotherapy [31, 33]. And, more recently, there are reports of BRAF MEKi. Similarly, few case reports have shown promising results after combination therapy with BRAF MEKi in PXA patients with *BRAF* mutations [12–14].

Unfortunately, tumors often develop resistance to targeted therapies, and hence, approaches to overcome resistance to BRAF MEKi would be very useful [22, 34]. One such approach is by inhibiting autophagy. Maddodi et al. showed that autophagy is triggered by hyperactivation of the ERK pathway by upstream *BRAF* activating mutations in melanomas in vitro and in vivo. [35] Autophagy inhibition in *BRAF* mutant melanoma animal inhibits tumor growth and prolongs survival [34]. In addition, high autophagic index in melanomas correlates with short survival and autophagy inhibition is effective in vitro. [36] Similar results are seen in *BRAF* V600E lung, and pancreatic and colorectal cancers, and hence, this is not tumor type specific [37, 16]. This strategy of combining autophagy inhibition with *BRAF* inhibition monotherapy in brain tumors was demonstrated in several brain tumors, including PXAs, using chloroquine [15–17]. Therefore, we combined BRAF MEKi with chloroquine and transformed a radiographically growing tumor (Fig. 4c, d) into a long (> 18 months) and sustained stability of disease in a patient without side effects for almost 1.5 years. This supports the hypothesis that autophagy inhibition can make brain tumors with *BRAF* mutations more chemosensitive to *BRAF* inhibition.

The current case report has several limitations, which include the lack of ability to generalize, risk of misinterpretation, and no established cause-effect relationship. As a single case report, findings cannot be generalized to represent similar groups of patients, partly for its dearth of an established cause–effect association from therapy, which can lead to misinterpretation. The observed response to treatment in this patient initially to dual BRAF MEKi, and subsequently to triple therapy with the addition of chloroquine, allows us to generate a hypothesis, aid in pharmacovigilance, and describe novel treatments when research designs are not possible due, for instance, to the rarity of the disease, or give us insight into the creation of controlled clinical trials in the future.

To our knowledge, this is the first case reported of combination therapy of BRAF MEKi with the autophagy inhibitor chloroquine in a brain tumor patient. This highlights the importance of a molecular interrogation of gliomas to provide an integrated diagnosis in gliomas and effective targeted treatment. Encouraged by these results, we reviewed glioma cases at Moffitt Cancer Center (MCC), who had similar molecular profiling, and found 3% patients with gliomas carrying *BRAF* mutations. These patients could potentially benefit from treatment with BRAF MEKi in combination with chloroquine. Molecular testing in neuro-oncology is providing new avenues of diagnosis and treatment, and detailed molecular interrogation should be considered routine.
